# Glial cells modulate retinal cell survival in rotenone-induced neural degeneration

**DOI:** 10.1038/s41598-021-90604-w

**Published:** 2021-05-27

**Authors:** Hiroshi Tawarayama, Maki Inoue-Yanagimachi, Noriko Himori, Toru Nakazawa

**Affiliations:** 1 Department of Ophthalmology, Tohoku University Graduate School of Medicine, 1-1 Seiryo-machi, Aoba-ku, Sendai, 980-8574 Japan; 2 Department of Retinal Disease Control, Tohoku University Graduate School of Medicine, Sendai, 980-8574 Japan; 3 Collaborative Program of Ophthalmic Drug Discovery, Tohoku University Graduate School of Medicine, Sendai, 980-8574 Japan; 4 Department of Advanced Ophthalmic Medicine, Tohoku University Graduate School of Medicine, Sendai, 980-8574 Japan; 5 Department of Ophthalmic Imaging and Information Analytics, Tohoku University Graduate School of Medicine, Sendai, 980-8574 Japan

**Keywords:** Neurological disorders, Cell death in the nervous system, Microglia, Neurotrophic factors, Retina, Neurodegeneration, Interleukins, Neurodegeneration

## Abstract

Administration of the mitochondrial complex I inhibitor rotenone provides an excellent model to study the pathomechanism of oxidative stress-related neural degeneration diseases. In this study, we examined the glial roles in retinal cell survival and degeneration under the rotenone-induced oxidative stress condition. Mouse-derived Müller, microglial (BV-2), and dissociated retinal cells were used for in vitro experiments. Gene expression levels and cell viability were determined using quantitative reverse transcription-polymerase chain reaction and the alamarBlue assay, respectively. Conditioned media were prepared by stimulating glial cells with rotenone. Retinal ganglion cells (RGCs) and inner nuclear layer (INL) were visualized on rat retinal sections by immunohistochemistry and eosin/hematoxylin, respectively. Rotenone dose-dependently induced glial cell death. Treatment with rotenone or rotenone-stimulated glial cell-conditioned media altered gene expression of growth factors and inflammatory cytokines in glial cells. The viability of dissociated retinal cells significantly increased upon culturing in media conditioned with rotenone-stimulated or Müller cell-conditioned media-stimulated BV-2 cells. Furthermore, intravitreal neurotrophin-5 administration prevented the rotenone-induced reduction of RGC number and INL thickness in rats. Thus, glial cells exerted both positive and negative effects on retinal cell survival in rotenone-induced neural degeneration via altered expression of growth factors, especially upregulation of microglia-derived Ntf5, and proinflammatory cytokines.

## Introduction

Rotenone, a natural compound found in the roots and stems of certain plants, acts as a strong inhibitor of nicotinamide adenine dinucleotide (NAD)-ubiquinone oxidoreductase of the mitochondrial respiratory chain^1,2^. Rotenone inhibits electron transfer from iron-sulfur centers in mitochondrial complex I to oxygen, resulting in excessive production of reactive oxygen species (ROS)^3,4^. Thus, rotenone administration has been used to elucidate the mechanisms underlying oxidative stress-induced neural degeneration following mitochondrial dysfunction^5–14^.

Chronic rotenone administration in rodents mimics the behavioral and pathological features observed in patients with Parkinson’s disease, such as loss of nigrostriatal dopaminergic neurons, the formation of Lewy body-like filamentous protein inclusions in degenerated neurons, and impaired locomotor activity^5,6,10–14^. A previous study reported retinal impairments preceding the loss of dopaminergic neurons in the substantia nigra of rotenone-administered rats; thus, retinal change is an early marker of Parkinson’s disease, at least in the rotenone-induced disease model^15^. Continuous intraperitoneal administration of rotenone results in elevated death of retinal ganglion cells (RGCs) and thinning of the swollen retina, probably owing to enhanced inflammation^15^. Furthermore, subcutaneous and intravitreal rotenone administration decreased the thickness of retinal layers, including the nerve layer, inner/outer nuclear layers, and inner plexiform layer, and fewer RGCs in rodents^7,8,16,17^. The putative mechanisms underlying rotenone-induced inner retinal degeneration have been described with an emphasis on neurons previously^7^.

Glial cells, including Müller cells and microglia, play numerous roles, namely, metabolism, cell debris removal, trophic factor release, and incorporation of excessive neurotransmitters, to maintain functional stability of retinal cells in normal and pathological conditions^18–21^. Thus, dysfunction of Müller cells and microglia is often concomitant with the development of retinal degeneration diseases owing to the loss of neuronal supporting activities^21–24^. Previous studies suggested that glial cells modulated retinal survival and inflammation through altered secretion of neurotrophic factors and inflammatory cytokines in pathological conditions^25–27^. Light irradiation, leading to photoreceptor cell death, upregulates growth factors, including fibroblast growth factor 2 (FGF2), nerve growth factor (NGF), glial cell line-derived neurotrophic factor (GDNF), and ciliary neurotrophic factor (CNTF) in microglia^25^. Moreover, the secretion of light-irradiated microglia upregulated brain-derived neurotrophic factor (BDNF), whereas it downregulated FGF2 in Müller cells^25^. Thus, glial secretion may modulate retinal cell survival during neural degeneration.

Since mitochondrial dysfunction-mediated oxidative stress contributes to the pathogenesis of retinal diseases, such as Leber’s hereditary optic neuropathy and glaucoma, rotenone administration provides an excellent model to study the mechanisms underlying oxidative stress-related retinal degeneration^9,28–30^. Additionally, the role of glial cells in rotenone-induced retinal degeneration remains elusive. Thus, to further characterize the rotenone-induced retinal degeneration model, we examined the roles of glia and their secretion in our study.

## Results

### Dose-dependent effects of rotenone on the survival of Müller and microglial cells

Mouse primary Müller cells and mouse BV-2 cells, instead of primary retinal microglia, were used to investigate the roles of glial cell secretion in rotenone-induced retinal degeneration since the number of retina-derived primary microglia was expected to be small to perform all our experiments. Past studies and our findings indicated that both primary microglia and BV-2 cells possessed the common properties to produce the same kinds of cytokines and growth factors (see the text below)^25,31–33^.

**Figure 1:**
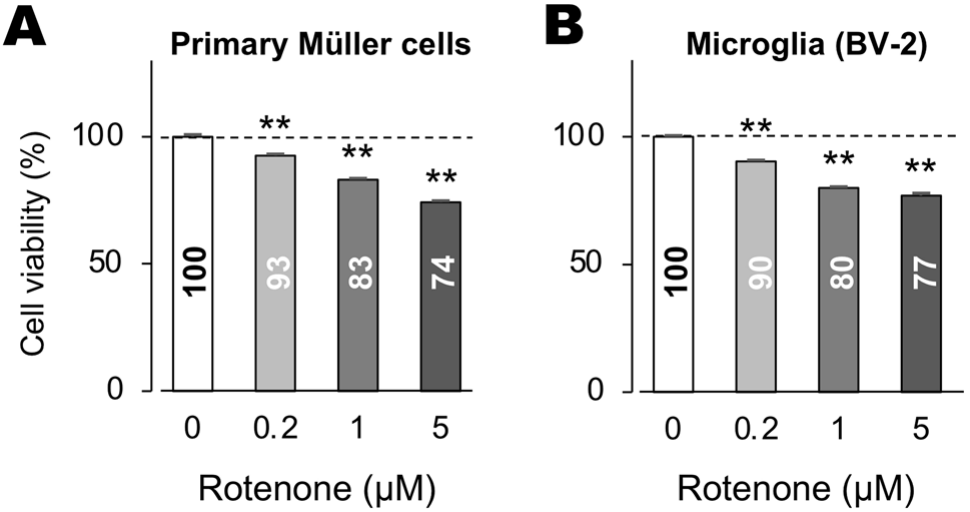
Viability of rotenone-treated Müller and BV-2 cells. (**A**, **B**) AlamarBlue cell viability assay of mouse primary Müller cells (**A**) and mouse brain-derived microglia BV-2 cells (**B**). Cell viability decreased in a dose-dependent manner for rotenone. Error bars indicate standard deviation. **P < 0.01 versus rotenone (−) controls (Dunnett’s test; *n* = 4).

We first monitored the dose-dependent effects of rotenone on survival of Müller and BV-2 cells using the alamarBlue assay; rotenone significantly decreased the viability of these cells in a dose-dependent manner (Fig. 1A,B).

### Rotenone-induced expression changes of growth factors and inflammatory cytokines in Müller and BV-2 cells

We then examined expression changes of growth factors, including *Ngf*, *Bdnf*, *Ntf3*, *Ntf5*, *Gdnf*, *Cntf*, and *Fgf2* genes, and inflammatory cytokines, including *interleukin* (*Il*)*1ß*, *Il8*, *C–C motif chemokine ligand 2* (*Ccl2*), and *tumor necrosis factor-alpha* (*Tnf-α*) genes, in Müller and BV-2 cells treated with various concentrations of rotenone using qRT-PCR.

**Figure 2:**
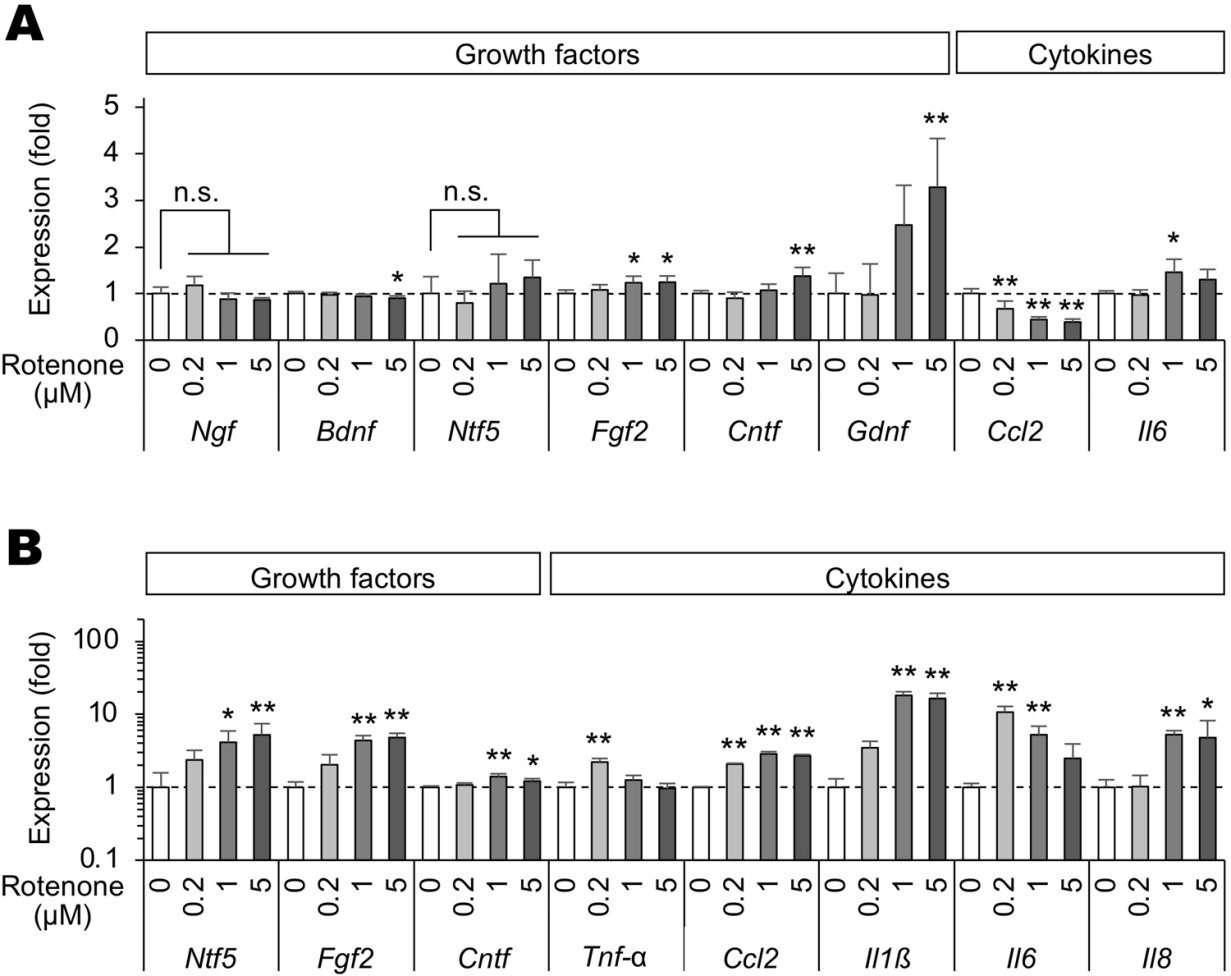
Rotenone-induced gene expression changes of growth factors and inflammatory cytokines in Müller and BV-2 cells. (**A**, **B**) Expression of *Ngf*, *Bdnf*, *Ntf5*, *Fgf2*, *Cntf*, *Gdnf*, *Ccl2*, and *Il6* genes in Müller cells (**A**) and expression of *Ntf5*, *Fgf2*, *Cntf*, *Tnf-α*, *Ccl2*, *Il1ß*, *Il6*, and *Il8* genes in BV-2 cells (**B**) treated with various concentrations of rotenone for 24 h. Expression levels were normalized against those of the *Gapdh* gene and were shown as values relative to those of each rotenone (−) control. Error bars indicate standard deviation. *P < 0.05, **P < 0.01 versus rotenone (−) control (Dunnett’s test; *n* = 4). n.s.: not significant.

Rotenone treatment increased the expression of *Fgf2*, *Cntf*, *Gdnf*, and *Il6* genes, whereas the expression of *Bdnf* and *Ccl2* genes decreased in Müller cells (Fig. 2A). No significant change was observed in the expression level of the *Ngf* gene (Fig. 2A). In contrast, rotenone treatment significantly increased the expression of *Ntf5*, *Fgf2*, *Cntf*, *Tnf-α*, *Ccl2*, *Il1ß*, *Il6*, and *Il8* genes in BV-2 cells (Fig. 2B). The expression level of *Il1ß*, *Tnf-α*, and *Ntf3* genes in Müller cells and the expression of *Ngf*, *Bdnf*, *Gdnf*, and *Ntf3* genes in BV-2 cells were lower than the detection limit (data not shown).

### Expression changes of growth factors and inflammatory cytokines in Müller and BV-2 cells stimulated with rotenone-treated cell conditioned media

We then examined the effects of glial secretion of rotenone-treated cells on gene expression of growth factors and inflammatory cytokines in Müller and BV-2 cells. Müller and BV-2 cells were cultured in media containing various concentrations of rotenone for 24 h, followed by culturing in fresh media for 24 more hours after washing rotenone-treated cells. Naïve Müller and BV-2 cells were cultured in conditioned media prepared from rotenone-treated BV-2 and Müller cells for 6 h, respectively, and the expression changes were analyzed using qRT-PCR.

**Figure 3:**
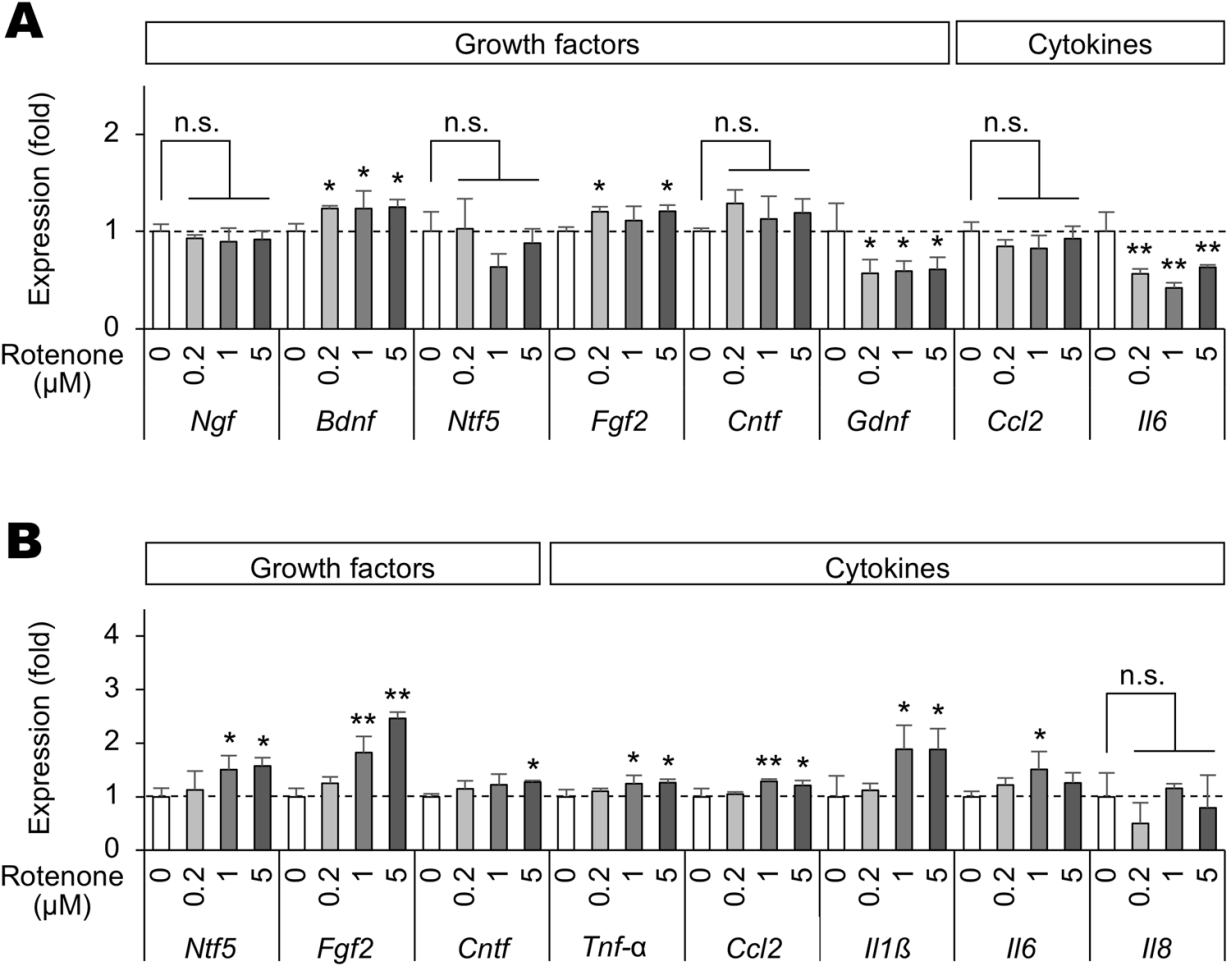
Rotenone-treated cell-conditioned media-induced gene expression changes of growth factors and inflammatory cytokines in Müller and BV-2 cells. (**A**, **B**) Expression of *Ngf*, *Bdnf*, *Ntf5*, *Fgf2*, *Cntf*, *Gdnf*, *Ccl2*, and *Il6* genes in Müller cells treated with conditioned media of BV-2 cells (**A**) and expression of *Ntf5*, *Fgf2*, *Cntf*, *Tnf-α*, *Ccl2*, *Il1ß*, *Il6*, and *Il8* in BV-2 cells treated with conditioned media of Müller cells (**B**) and stimulated with various concentrations of rotenone for 6 h. Expression levels were normalized against those of the *GAPDH* gene and were shown as values relative to those of each rotenone (−) control. Error bars indicate standard deviation. *P < 0.05, **P < 0.01 versus rotenone (−) control (Dunnett’s test; *n* = 4). n.s.: not significant.

Conditioned media prepared from rotenone-stimulated BV-2 cells increased the expression level of *Bdnf* and *Fgf2* genes in Müller cells, whereas the expression level of *Gdnf* and *Il6* genes decreased (Fig. 3A). It did not affect the expression level of *Ngf*, *Ntf5*, *Cntf*, and *Ccl2* genes (Fig. 3A). In contrast, conditioned media prepared from rotenone-stimulated Müller cells increased the expression level of *Ntf5*, *Fgf2*, *Cntf*, *Tnf-α*, *Ccl2*, *Il1ß*, AND *Il6* genes in BV-2 cells but did not affect *Il8* expression level (Fig. 3B).

### Effects of rotenone-induced glial cell secretion on oxidative stress-induced death of dissociated retinal cells

**Figure 4:**
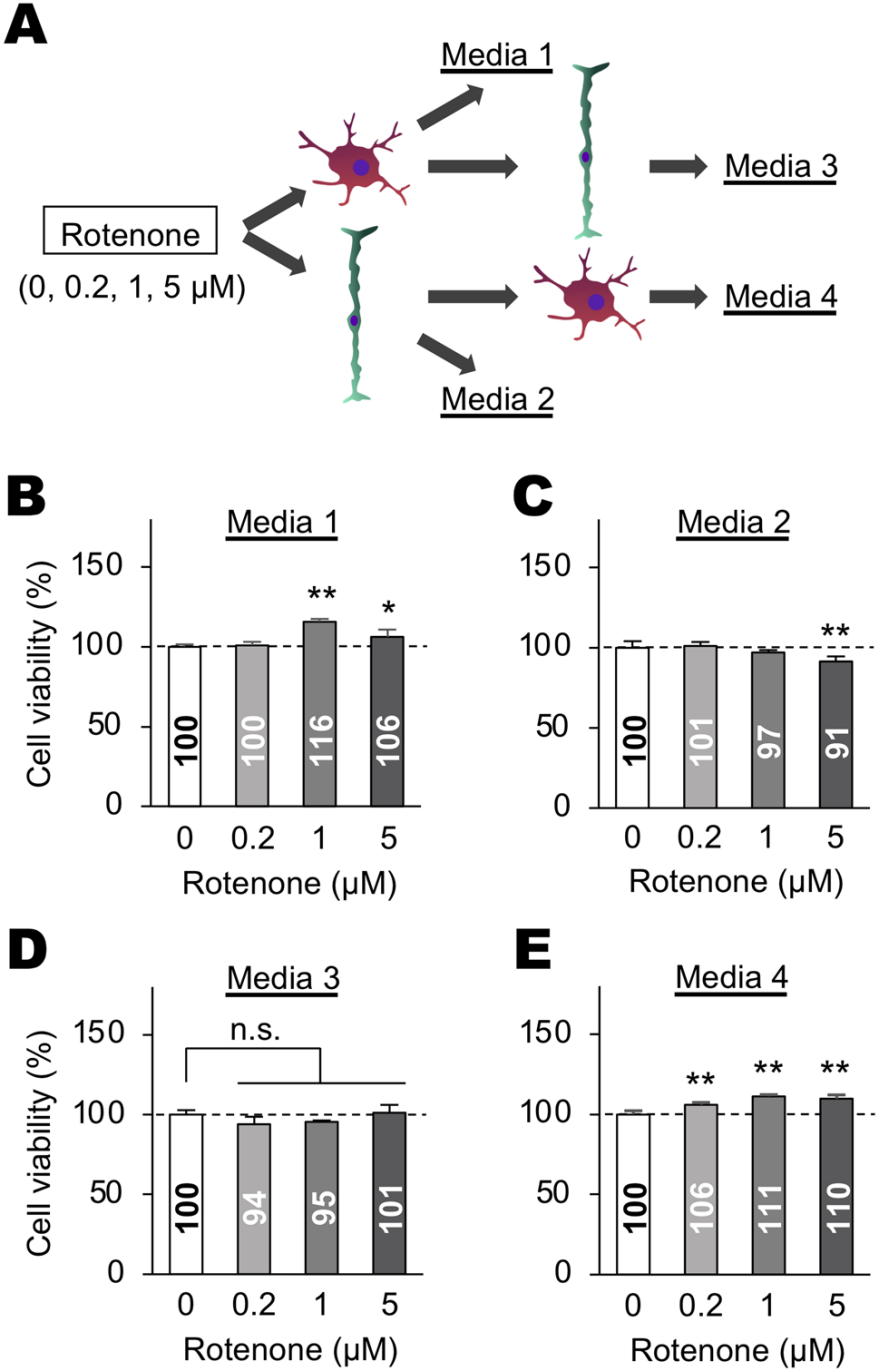
Effects of conditioned media of rotenone-treated Müller and BV-2 cells on survival of dissociated retinal cells. (**A**) Schematic for the preparation of conditioned media of rotenone-treated Müller and BV-2 cells. BV-2 and Müller cells were treated with various concentrations of rotenone for 24 h and were then cultured with fresh media for 24 h more. The conditioned media of BV-2 and Müller cells were collected (Media 1 and 2, respectively). Naïve Müller and BV-2 cells were cultured with Media 1 and 2 for 24 h and were then collected (Media 3 and 4, respectively). (**B**–**E**) Cell viability assay on mouse dissociated retinal cells cultured with Media 1–4 for 24 h, respectively. Viability was shown as values relative to that of rotenone (−) control. Error bars indicate standard deviation. *P < 0.05 and **P < 0.01 versus rotenone (−) control (Dunnett’s test; *n* = 4). n.s.: not significant.

We then examined the effects of secreted molecules from rotenone-stimulated cells and cells stimulated with rotenone-treated cell conditioned media on oxidative stress-induced death in dissociated retinal cells. Conditioned media prepared in different glial cell combinations presented in Fig. 4A (Media 1–4) were used for the experiment (see Materials and methods for details). Dissociated mouse retinas were cultured in media 1 (rotenone-treated BV-2 cell-conditioned media), Media 2 (rotenone-treated Müller cell-conditioned media), Media 3 (Media 1-treated Müller cell-conditioned media), and Media 4 (Media 2-treated BV-2 cell-conditioned media). Cell viability was then determined using the alamarBlue assay. Media 1 and 4 significantly increased cell viability in dissociated retinal cell cultures (Fig. 4B,E), whereas Media 2 decreased cell viability (Fig. 4C). In contrast, Media 3 did not affect cell viability (Fig. 4D).

### Intravitreal Ntf5 administration inhibited rotenone-induced retinal cell death

**Figure 5:**
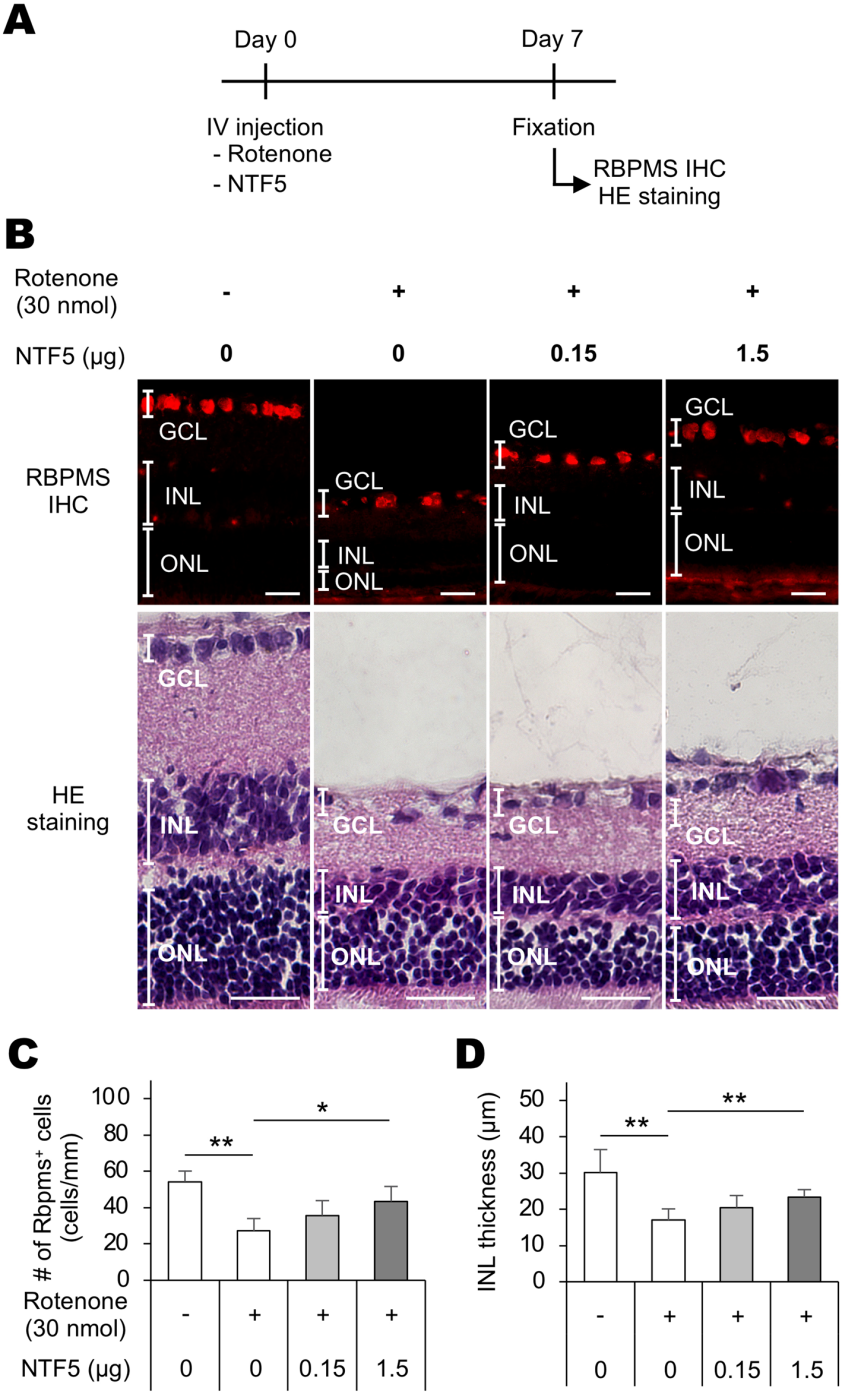
Effects of intravitreal administration of NTF5 proteins on rotenone-induced retinal degeneration in rats. (**A**) Schematic of the experimental procedure. (**B**) RBPMS immunohistochemistry (IHC) and hematoxylin/eosin (HE) staining of retinal sections prepared from animals intravitreally treated with rotenone and NTF5 recombinant proteins. (**C, D**) Quantification of the number of RBPMS^+^ RGCs (**C**) and thickness of the INL in rotenone- and NTF5-administrated rats. RBPMS, RNA-binding protein with multiple splicing; RGC, retinal ganglion cell. Scale bars: 50 µm.

Media 1 and 4 increased the survival of dissociated retinal cells (Fig. 4), and both stimulated *Ntf5* expression in BV-2 cells (Figs. 2B, 3B). Thus, to examine the in vivo effects of NTF5 on the rotenone-induced loss of retinal cells, rats were intravitreally administered with rotenone (30 nmol) and NTF5 recombinant proteins (0.15 or 1.5 µg), and RBPMS immunohistochemistry and HE staining were performed on retinal sections seven days later (Fig. 5A). Intravitreal rotenone administration resulted in fewer RBPMS^+^ RGCs and thinner INL (Fig. 5B), which were consistent with previous findings^7^. However, NTF5 recombinant proteins attenuated rotenone-induced retinal impairment in a dose-dependent manner (Fig. 5C,D).

## Discussion

**Figure 6:**
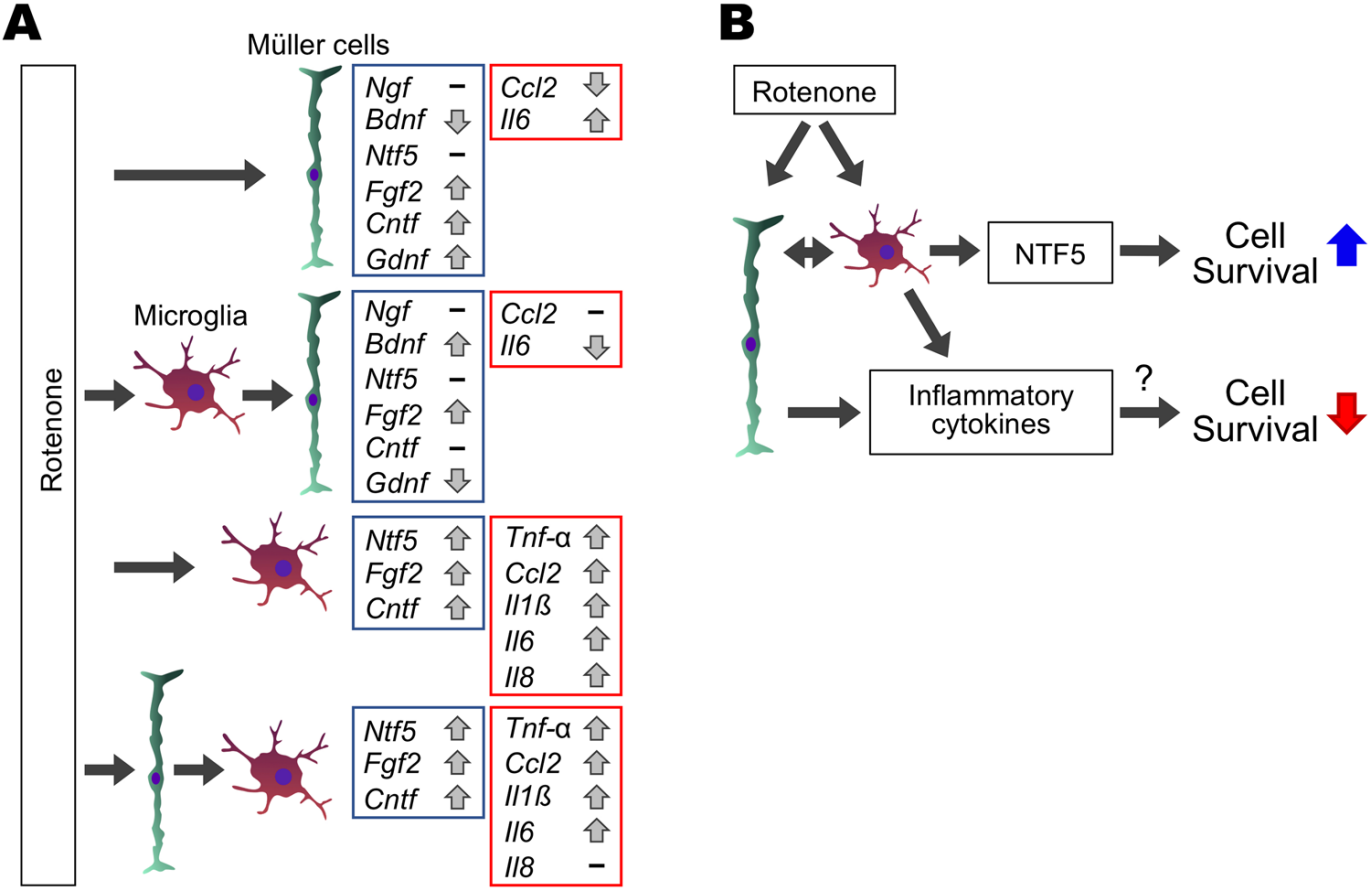
Schematic diagram explaining glial roles in rotenone-induced retinal degeneration. (**A**) Summary of expression changes in growth factors and inflammatory cytokines in Müller and BV-2 cells stimulated with rotenone or rotenone-stimulated cell-conditioned media. Gene expression changes of growth factors and inflammatory cytokines were shown in the boxes colored blue and red, respectively. (**B**) Positive and negative effects of rotenone-stimulated glial cells on retinal survival. Expression of inflammatory cytokines is upregulated in rotenone-treated Müller and microglial cells. These would result in a decreased survival of retinal cells owing to excessive inflammatory responses. In contrast, microglia activate to produce NTF5 in response to rotenone or secretion of rotenone-treated Müller cells, which leads to increased survival in retinal cells owing to its neuroprotective activity.

The present study indicated that administration of mitochondrial complex I inhibitor rotenone or rotenone-stimulated cell-conditioned media altered gene expression of these molecules in glial cells, as summarized in Fig. 6A. This altered expression would potentially modulate retinal cell survival (Fig. 6B). Consistent with the present findings, it has been shown that neurotoxic stimuli and the following glial cell secretions activated the production of not only proinflammatory cytokines but also growth factors in Müller and microglial cells^25,34–39^. Some studies reported the mechanisms underlying the glial secretion-mediated alteration of growth factor gene expression in other glia: Microglia-derived NGF downregulates the *Fgf2* expression in cultured Müller cells via activation of a common neurotrophin receptor p75 under oxidative stress conditions^25,40^. This leads to enhanced cell death of photoreceptors due to the decrease of the FGF2-mediated neuroprotective activity^25,40^. Similarly, dysregulated expression of growth factors and inflammatory cytokines in glial cells and its secondary effects via the glial secretions could modulate cell survival and death in the rotenone-induced retinal degeneration.

Neuroprotective effects of NTF5 have been reported previously^41–44^. Retinal cells displayed higher susceptibility to ischemic injuries in *Ntf5*-deficient animals than wild-type animals^43^. RGCs were more resistant to cell death following optic nerve crush in alpha-lipoic acid-administered rats where *Ntf5* expression was upregulated in RGCs^44^. Furthermore, NTF5 could prevent naturally occurring retinal cell death during development^42,43^. Since our findings indicated that (1) *Ntf5* expression was upregulated in BV-2 cells in response to rotenone and rotenone-stimulated Müller cell-conditioned media, (2) conditioned media obtained from these BV-2 cell cultures (Media 1 and 4, Fig. 4A) promoted the viability of dissociated retinal cells, and (3) intravitreal Ntf5 administration attenuated rotenone-induced retinal degeneration, we concluded that microglia exerted retinal cell survival-promoting activity by upregulating NTF5 expression.

It is known that not only NTF5 but also BDNF preferentially utilize the tropomyosin-related kinase (Trk) B receptor, a member of the neurotrophic tyrosine kinase receptor family, as a signal transducer, suggesting that BDNF plays identical roles to NTF5 in regulating retinal cell survival^45–47^. Supporting this, past studies reported the neuroprotective effect of BDNF on retinal cells in animal glaucoma models and hypoxia- and glucose deprivation-induced injury models^48–50^. However, in our study, media prepared from Müller cells treated with rotenone-stimulated BV-2 cell-conditioned media (Media 3, Fig. 4A) had no effects on the viability of dissociated retinal cell cultures, although it induced *Bdnf* upregulation in Müller cells (Fig. 3A). NTF5 and BDNF had similar but distinguishable roles in synaptic transmission, neuronal survival, and growth in a context-dependent manner^51–54^. Additionally, it has been reported that NTF5 and BDNF display different binding properties to the TrkB receptor^52,55^. Thus, the differences in molecular properties between NTF5 and BDNF might explain the reason conditioned media rich in NTF5 but not rich in BDNF promoted retinal cell survival in our study. Alternatively, the increasing rate of *Bdnf* upregulation in Müller cells, stimulated with BV-2-conditioned media (Media 3), was too less to promote the viability of dissociated retinal cells, or Müller cells produce toxic molecules to decrease retinal cell survival (Fig. 4C), which antagonize the cell survival-promoting activity of BDNF.

Our in vivo study demonstrated that intravitreal administration of NTF5 attenuated the rotenone-induced loss of RGCs and INL cells in a dose-dependent manner. Given that the TrkB receptor is expressed in RGCs and amacrine cells in the ganglion cell and inner nuclear layers, NTF5 could rescue rotenone-induced cell death of these cells by its direct effect through TrkB-mediated signaling^56,57^. Moreover, NTF5 might be implicated in the prevention of rotenone-induced retinal cell death in an indirect manner, i.e., through the activation of Müller cells expressing TrkB and downregulation of other growth factors secreted from glia similar to FGF2-mediated BDNF downregulation described above^25,40,58^. Both the direct and indirect effects of microglia-derived NTF5 could play pivotal roles in the modulation of survival in various types of retinal cells despite the TrkB expression. Although we have not focused on the impact of astrocytes on rotenone-induced retinal degeneration in this study, astrocytes also contribute to the promotion of retinal cell survival; the neuroprotective effects of astrocytes in injured retinal tissues has been demonstrated by previous studies^59^.

Our study indicated that treatment with rotenone or rotenone-stimulated Müller cell-conditioned media significantly increased the expression level of *Il1ß* and *Il6* in BV-2 cells. These cytokines exert overlapping and synergistic activities to stimulate the production of other inflammatory cytokines^60–63^. Thus, IL1ß and IL6 would exacerbate an inflammation state, leading to excessive neural cell death. In contrast, some studies have demonstrated the positive effects of IL1ß and IL6 on retinal cell survival^64,65^. Intravitreal administration of IL1ß or IL6 resulted in microglial activation and neural survival promotion in N-methyl-D-aspartate (NMDA)-treated retinas^64,65^. Deficiency of IL1ß receptor (IL1R1) and microglial ablation diminished the survival-promoting effects of these cytokines^64,65^. However, administration of NMDA prior to IL6 administration resulted in exacerbation of NMDA-induced retinal degeneration^64^. These findings suggested that proinflammatory cytokines such as IL1ß and IL6 secreted from activated microglia exerted positive and negative effects on retinal cell survival in normal and pathological conditions, respectively. Thus, increased expression of proinflammatory cytokines may result in enhanced neural cell death in rotenone-treated retinas.

In summary, we investigated the potential roles of glial cells and their secretion in rotenone-induced retinal degeneration reflecting the pathology of mitochondrial oxidative stress-induced retinal diseases in this study^8,9,16^. The obtained results suggested that the microglia-derived NTF5 prevents rotenone-induced retinal cell death. Thus, adeno-associated virus-based constitutive expression of exogenous NTF5 in the microglia or intravitreal administration of exogenous NTF5 would be effective in treating oxidative stress-associated retinal degeneration diseases.

## Methods

### Animals

C57BL/6 male and female mice (8–10 weeks old) and Sprague–Dawley (SD) male rats (8–10 weeks old) were purchased from SLC (Shizuoka, Japan) and maintained at animal facilities in Tohoku University Graduate School of Medicine (Sendai, Japan) under a 12-h light/dark cycle. Mice were mated to obtain pups for primary Müller cell preparation. Male mice were used for retinal dissociation culture experiments. All experimental procedures conformed to "Regulations for Animal Experiments and Related Activities at Tohoku University" and were reviewed by the Institutional Laboratory Animal Care and Use Committee of Tohoku University, and finally approved by the President of University. This study was performed in compliance with the ARRIVE guidelines.

### Preparation of mouse primary Müller cells

Preparation and culture of Müller cells have been described previously^25,66^. Briefly, eyes dissected from postnatal day (P)5 to P8 mouse pups were incubated in Dulbecco’s modified Eagle medium (DMEM; Thermo Fisher Scientific, Waltham, MA, USA) containing 10% fetal bovine serum (FBS; Thermo Fisher Scientific) at room temperature overnight. After washing with Dulbecco's phosphate buffered saline, eyes were treated with 0.25% trypsin solution for 15 min. Retinas were isolated from eyes using sharp forceps and were broken into small pieces by pipetting several times. Prepared retinal explants were cultured in DMEM containing 10% FBS (DMEM10) in a CO_2_ incubator at 37 °C. Growing Müller cells out of the explants were sub-cultured upon reaching 80% confluency using a trypsin solution. Primary Müller cells with high passage numbers displayed drastic growth inhibition and morphological change. Thus, Müller cells passaged less than 6 times were used for the in vitro experiments. Müller cell cultures are shown in the supplementary figure. We identified Müller cells based on their characteristic shape that helped us distinguish them from astrocytes^67^. Nearly all of the cultured cells displayed the morphological features of Müller cells.

### Conditioned media preparation of rotenone-treated Müller cells and microglia

Mouse primary Müller cells, prepared as described previously, and mouse brain-derived microglia (BV-2) were maintained in DMEM10. Müller and BV-2 cells were seeded at 3.0 × 10^5^ and 6.5 × 10^5^ cells in 60 mm dishes, respectively, and cultured in media containing various concentrations of rotenone (Tokyo chemical industry, Tokyo, Japan) for 24 h. Cells were washed with DMEM10 twice to remove rotenone and were then cultured in fresh media for an additional 24 h. Culture media were collected and passed through a 0.22 µm filter (Merck Millipore, Burlington, MA, USA). To obtain double-conditioned media, Müller and BV-2 cells (1.4 × 10^5^ and 3.0 × 10^5^ cells, respectively) were cultured with rotenone-stimulated BV-2 and Müller cell-conditioned media (3 mL) in 35 mm dishes for 24 h, respectively.

### Quantitative reverse transcription-polymerase chain reaction (qRT-PCR)

Primary Müller cells and BV-2 cells were seeded at 0.5 × 10^4^ and 5 × 10^4^ cells/well in 96-well cell culture plates, respectively, and were treated with various concentrations of rotenone for 24 h. In some cases, these cells were treated with conditioned media instead of rotenone containing media for 6 h. After cell lysis in each well of the 96-well cell culture plates, RNA was reverse transcribed into cDNA using the SuperPrepII cell lysis & RT kit (Toyobo, Osaka, Japan) according to the manufacturer’s instruction. qRT-PCR was performed in a 7500 fast real-time PCR system (Thermo Fisher Scientific) using TaqMan fast universal PCR master mix (Thermo Fisher Scientific) and a mixture of predesigned TaqMan primers and probes [Thermo Fisher Scientific or Integrated DNA Technologies (Coralville, IA, USA)] (see Table 1). Gene expression was examined in 4 individual wells of the 96-well plates per each treatment (*n* = 4), and the average and standard deviation were subsequently calculated. The gene expression level is shown as a percentage of rotenone-untreated controls.

**Table 1:** List of the primer and probe mixtures used for quantitative qRT-PCR.

Species	Genes	Supplier	Assay ID
mouse	*Gapdh*	Integrated DNA Technologies	Mm.PT.39a.1
mouse	*Ngf*	Integrated DNA Technologies	Mm.PT.58.14181538
mouse	*Bdnf*	Integrated DNA Technologies	Mm.PT.58.8157970
mouse	*Ntf5*	Integrated DNA Technologies	Mm.PT.58.11243625
mouse	*Fgf2*	Integrated DNA Technologies	Mm.PT.56a.5129235
mouse	*Cntf*	Integrated DNA Technologies	Mm.PT.58.32700675.g
mouse	*Gdnf*	Integrated DNA Technologies	Mm.PT.58.6003912
mouse	*Tnf-α*	Integrated DNA Technologies	Mm.PT.58.12575861
mouse	*Ccl2*	Integrated DNA Technologies	Mm.PT.58.42151692
mouse	*Il-1ß*	Integrated DNA Technologies	Mm.PT.58.41616450
mouse	*Il-6*	Integrated DNA Technologies	Mm.PT.58.10005566
mouse	*Il-8*	Integrated DNA Technologies	Mm.PT.58.9981538

### Retinal dissociation culture

Retinal dissociation cultures were performed as described previously^68^. Briefly, 6 retinas were dissected from 3 male mice (8–10 weeks old) and dissociated into single cells using the neural tissue dissociation kit (Miltenyi Biotec, Bergisch Gladbach, Germany). Dissociated retinal cells were mixed and resuspended in Neurobasal-A medium (Thermo Fisher Scientific) containing B-27 supplement without antioxidants (Thermo Fisher Scientific). Cells were seeded at 1 × 10^5^ cells/well (50 µL) in 96-well cell culture plates. One hour after seeding, the same volume of Müller and/or BV-2 cell-conditioned media was added to retinal cell mixtures and cultured for 24 h.

### Cell viability assay

Primary Müller cells, BV-2 cells, and dissociated retinal cells were seeded at 0.5 × 10^4^, 5 × 10^4^, and 1 × 10^5^ cells/well in 96-well cell culture plates, respectively, and were incubated in DMEM10 containing various concentrations of rotenone or Müller and/or BV-2 cell-conditioned media for 24 h. Culture media were removed, and cells were then incubated in DMEM10 containing 10% of the alamarBlue cell viability reagent (Thermo Fisher Scientific) for 3 h. In the case of retinal dissociated cells, alamarBlue was added to cultures one hour after seeding. Fluorescence intensity was measured at 590 nm (excitation: 560 nm) using a SpectraMax M2e microplate reader (Molecular Devices, San Jose, CA, USA). The alamarBlue signals were examined in 4 individual wells of 96-well plates per each treatment (*n* = 4), and the average and standard deviation were subsequently calculated. The viability is shown as a percentage of the rotenone-untreated controls. The same experiments using the cell cultures prepared from different individuals were performed twice to confirm the obtained results.

### Intravitreal administration

SD rats were anesthetized using intraperitoneal injection of 8 mg/kg xylazine (Bayer Yakuhin, Osaka, Japan) and 80 mg/kg ketamine (Daiichi Sankyo, Tokyo Japan). A mixture of 2.5 µL of rotenone dissolved in dimethyl sulfoxide (30 nmol) and/or the same volume of neurotrophin (NTF)5 recombinant proteins (0.15 or 1.5 µg) dissolved in Ca^2+^ and Mg^2+^-free Dulbecco's phosphate buffered saline (DPBS; 10 mM, pH 7.4) were administered intravitreally using a micro syringe with a 32-G sharp needle (Ito corporation, Shizuoka, Japan). DPBS was used as an injection control.

### Histology

Eyes were dissected from SD rats seven days after intravitreal injection of rotenone alone or together with Ntf5 recombinant proteins and were fixed with 4% paraformaldehyde in PBS overnight at 4 °C. Fixed eyes were embedded in optimal cutting temperature compound (Sakura Finetek Japan, Tokyo, Japan) and were cut into 10 µm-thick sections using a cryostat CM3050S (Leica Biosystems, Wetzlar, Germany). Immunostaining of retinal sections was performed as reported before^69^. Briefly, retinal sections were treated with blocking solution (10% normal donkey serum in PBS containing 0.01% Tween 20), followed by treatment with rabbit primary antibody (Abcam, Cambridge, UK; 1:500 dilutions with blocking solution) for RNA-binding protein with multiple splicing (RBPMS), which is a selective marker for retinal ganglion cells^70^. After extensive washing, retinal sections were incubated with Cy3-conjugated anti-rabbit IgG antibody (Jackson ImmunoResearch, West Grove, PA, USA; 1:500 dilutions with blocking solution) and 4′,6-diamidino-2-phenylindole (DAPI; Dojindo, Kumamoto, Japan). To visualize the inner nuclear layer (INL) cells, retinal sections were stained with hematoxylin and eosin (HE; Muto Pure Chemicals, Tokyo, Japan).

### Quantification of the retinal ganglion cell number and the INL thickness

Photos were acquired using a fluorescent microscope BZ-X810 (Keyence, Osaka, Japan) with a 20 × objective lens on microscopic fields 200 µm far from the optic nerve head of at least three retinal sections stained with RBPMS antibodies or HE per eye. The number of RBPMS^+^ RGCs and INL thickness in each image were quantified using ImageJ software (National Institute of Health, Bethesda, MD, USA). The average was calculated from five individual eyes per each experimental group.

### Statistical analyses

Quantitative data were analyzed using the Tukey–Kramer and Dunnett’s tests with JMP Pro 14 software (SAS Institute, Cary, NC, USA). A *P*-value of < 0.05 was considered statistically significant.

## Supplementary Information

Supplementary Information.

## Data Availability

The datasets used and analyzed in the current study are available from the corresponding author upon request.
