# Supplementary material for: Glial cells modulate retinal cell survival in rotenone-induced neural degeneration
Source: Sci Rep. 2021 May 27;11:11159. doi: 10.1038/s41598-021-90604-w (PMC8159960; doi:10.1038/s41598-021-90604-w)
Supplement: Supplementary file 1 — Supplementary Information. [file 41598_2021_90604_MOESM1_ESM.doc]

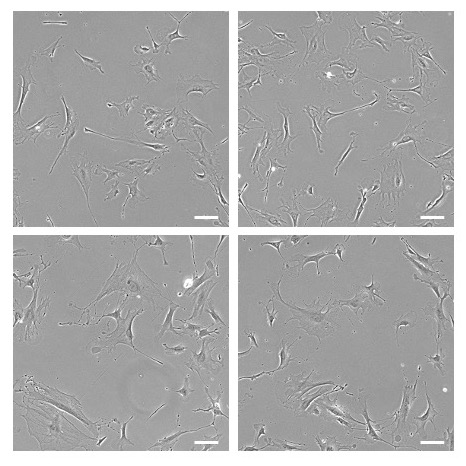


**Supplementary figure.** Phase-contrast images of mouse retina-derived Müller cell cultures. Müller cell cultures were prepared as described in the methods of the text and passaged 6 times. Photos were acquired using a microscope BZ-X810 (Keyence, Osaka, Japan) with a 20× objective lens, and 4 different microscopic fields were shown. Scale bars: 100 µm.
